# Supplementary material for: The effect of intrapartum prolonged oxygen exposure on fetal metabolic status: secondary analysis from a randomized controlled trial
Source: Front Endocrinol (Lausanne). 2023 Jun 27;14:1204956. doi: 10.3389/fendo.2023.1204956 (PMC10335765; doi:10.3389/fendo.2023.1204956)
Supplement: Supplementary file 3 [file DataSheet_3.zip › Result-X101SC21092977-Z01-J001-B1-42/1.MetQuant-QC/Samples_pos-pcaloading.pdf]

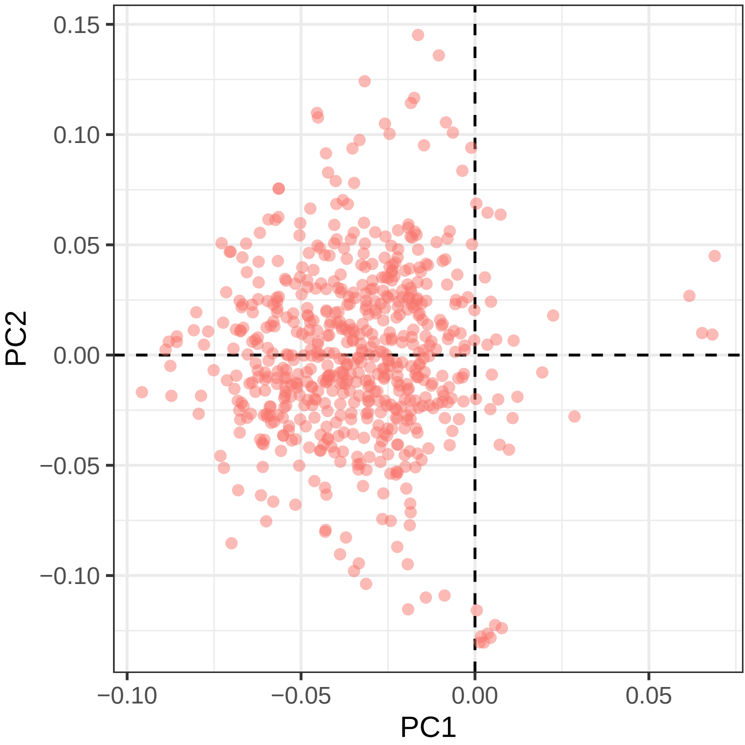

0.15
0.10
0.05
0.00
−0.05
−0.10
PC2
−0.10
−0.05
0.00
0.05
PC1
